# History and phylogeny of intermediate filaments: Now in insects

**DOI:** 10.1186/1741-7007-9-16

**Published:** 2011-02-28

**Authors:** Harald Herrmann, Sergei V Strelkov

**Affiliations:** 1 Group Functional Architecture of the Cell (B065) German Cancer Research Center (DKFZ) Im Neuenheimer Feld 280 D-69120 Heidelberg, Germany; 2 Faculteit Farmaceutische Wetenschappen Katholieke Universiteit Leuven Herestraat 49 Bus 822 B-3000 Leuven, Belgium

## Abstract

Intermediate filaments include the nuclear lamins, which are universal in metazoans, and the cytoplasmic intermediate filaments, which are much more varied and form cell type-specific networks in animal cells. Until now, it has been thought that insects harbor lamins only. This view is fundamentally challenged by the discovery, reported in *BMC Biology*, of an intermediate filament-like cytoplasmic protein, isomin, in the hexapod *Isotomurus maculatus*. Here we briefly review the history of research on intermediate filaments, and discuss the implications of this latest finding in the context of what is known of their structure and functions.

See research article: http://www.biomedcentral.com/1741-7007/9/17

## *From hair to nuclear organization*

Biological specimens enriched in intermediate filament (IF) proteins were among the first to be placed into an x-ray beam for structural analysis, back in the 1930s by William Astbury. He used hair, in its non-stretched and stretched form, rightly deducing that such extended, stable and flexible rods are made from highly ordered proteins. However, it took the thesis work of a graduate student several years later to explain fully these first, surprisingly simple diffraction patterns. The student was Francis Crick. He realized that the keratin α-helices in hair are packed as “simple coiled coils”, remarking later, in his 1988 autobiography, that at that time “helices were in the air”. This excitement was partly due to the discovery of the α-helical fold by Linus Pauling and his group as a fundamental structural principle embodied in the muscle proteins myosin and tropomyosin; and in the years that followed, more α-helix-rich proteins were discovered and grouped together as fibrous proteins. Years later the excitement of biochemists was gone. The second edition of classical textbook *Biological Chemistry* by Mahler and Cordes (1971) lists them simply under scleroproteins together with collagen and gelatin, without further mention.

**Figure 1:**
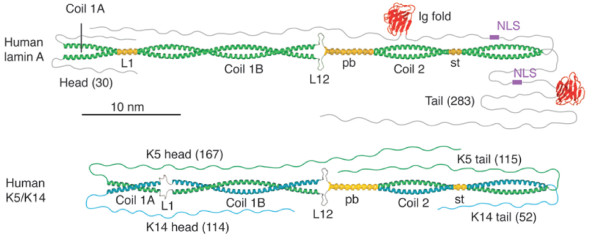
**All intermediate filaments have essential structural features in common.** A schematic molecular model of a coiled-coil dimer is shown for human lamin A (upper part) and human keratins 5 and 14, which heterodimerize to assemble into the keratin filament (lower part). The two molecular complexes are aligned with respect to coil 2. NLS, nuclear localization signal; pb, paired bundle; L1, linker L1; L12, linker L12; st, stutter (adapted from [10]).

This scenario changed when in 1968 the group of Howard Holtzer discovered IFs as a further independent filament system in cells obtained from chicken muscle in addition to the well established actin and myosin filaments, highly abundant in myocytes. By conventional electron microscopy, the diameter of these new filaments was determined to be intermediate between that of actin and myosin filaments, hence intermediate filaments or 10-nm filaments (see [1]). Within the next 10 years these new filaments were found in all vertebrate tissues and cultured cell lines investigated, and in many other animals too; and subsequently, the massive DNA sequencing efforts of the 1980s produced two major insights into this protein class. First, IF proteins from various tissues all exhibit a conserved central α-helical rod domain, organized so that two chains can form a parallel in-register coiled coil (Figure 1; [1]]), and  which is flanked by non-α-helical domains of very different character and size. Second, they are only found in metazoan species and appear to be absent from plants and fungi.

It came as a great surprise when the cell nucleus was found to contain fibrillar substructures - the nuclear lamina - composed of specialized IF proteins, the lamins. As so often in science, these entities, discovered last, turned out to be the evolutionary ancestors of the whole intermediate filament multigene family. Simple metazoans such as *Hydra attenuata* were found to express at least nuclear IF proteins, and a comparative analysis of their lamin sequences and the other known lamin and IF protein sequences led to the conclusion that IFs originated in an ur-lamin [2]. The simple invertebrate *Caenorhabditis elegans*, which has a single nuclear lamin, also harbors eleven genes coding for cytoplasmic IF proteins, four of which have been demonstrated to be essential for viability [3]; but the fruit fly *Drosophila melanogaster*, which expresses the two nuclear lamins -  lamin A and lamin B - characteristic of mammalian species, does not exhibit any cytoplasmic IF protein. This led to the conclusion that insects lack cytoplasmic IFs - a conclusion that is now challenged by Mencarelli *et al. *[4], who detected abundant cytoplasmic structures in the mid-gut cells of the hexapod *Isotomurus maculatus* (commonly known as the springtail), and have isolated the protein, cloned the DNA from the deduced sequence, compared the sequence with those of known IFs, reassembled filaments from the expressed protein *in vitro*, and conclude that the protein, which they call isomin, is an intermediate filament protein. 

Before discussing the implications of this discovery, we need to ask what are the defining features of an intermediate filament protein, and how does isomin fit the definition?

All intermediate filament proteins are coiled-coil proteins, but not all coiled-coil proteins are intermediate filament proteins. The conclusion that IF proteins are absent from plants and fungi for example rests on the basis of the fully sequenced genomes of the thale cress (*Arabidopsis thaliana*) and of bakers’ yeast (*Saccharomyces cerevisiae*); but coiled coil proteins are quite abundant in these organisms, in myosins, kinesins and tropomyosins as well as in transcription factors and in the structural maintenance of chromosomes proteins (SMCs), which contain extended coiled coils and are found in all cells. Even bacterial cells are assumed to contain some extended coiled-coil proteins [5], and notably, a bacterial protein essential for the organization of cell curvature, crescentin, has been described to exhibit many features that are found in IF proteins, and hence has been termed IF-like [6]. So how are coiled-coil proteins recognized, and how are IF proteins distinct?

## *What makes a* bona fide *intermediate filament?*

Coiled-coil forming parts within protein sequences can be identified relatively easily by looking for heptad repeats - amino-acid sequence motifs that, first, allow the formation of an α-helix and second, have apolar residues periodically at positions a and *d* within the seven amino-acid repeat (*abcdefg*)_n_. It is the long-range regular disposition of hydrophobic residues that forces two α-helices into a superhelix, that is, a coiled-coil dimer, and their hydrophobic character also determines the strength of the interaction of the α-helices within the coiled coil [7]. Charged amino acids within the coiled coil serve as ‘trigger motifs’, essential for dimerization [1].

What then distinguishes intermediate filament proteins from other coiled-coil forming proteins? Although many proteins follow a common fold without pronounced similarity in primary amino acid sequence, there are conserved “consensus sequences” about 20 amino acids long in IF proteins, and the organization of the central α-helical rod domain is followed quite strictly in IF proteins: the individual segments of coiled-coiled forming parts are separated by short variable linker motifs and exhibit a conserved number of amino acids (coil 1A: 35; coil 1B: 101; coil 2: 142; see Figure 1). A notable peculiarity of nuclear IF proteins is that they have six heptads, or 42 amino acids, more in coil 1B than vertebrate cytoplasmic IF proteins. Notably, invertebrate cytoplasmic proteins still have the long coil 1B, except for some tunicates - that is, chordates that are however still invertebrates. All these proteins have a very similar organization of coil 2, which carries in the middle region a so-called stutter region, an irregularity in the heptad pattern, at the very same position relative to IF consensus motif 2.

**Figure 2:**
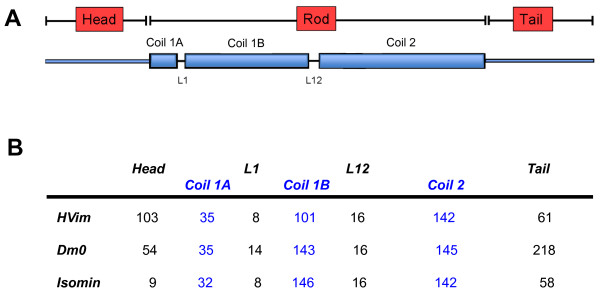
**Relationship of isomin domain organization to that of conventional IF proteins. (a)** Schematic model for the domain organization of an IF protein. Boxes: helical domains with coiled-coil forming propensity. Lines: non-α-helical domains. **(b)** Number of amino acids harbored by individual subdomains. HVim: human vimentin; Dm0: Drosophila melanogaster lamin D_m0_. The isomin sequence was analysed using NetSurfP server (Protein Surface Accessibility and Secondary Structure Predictions, Technical University of Denmark).

As mentioned above, up to now it has been thought, because of the absence of cytoplasmic IF proteins from *Drosophila melanogaster*, that insects do not have cytoplasmic intermediate filaments. This notion has now been challenged by the discovery of a protein in the hexapod *Isotomurus maculatus*, that resembles cytoplasmic IF proteins from other invertebrates quite distinctly [4]. When the primary sequence of isomin is aligned to a *Drosophila*  lamin D_m0_ (the B-type lamin), coil 1B and coil 2 match quite well in length (Figure 2). For coil 1A the number of amino acids qualifying for an α-helical fold is somewhat lower than the conventional 35 amino acids of coil 1A in standard IF proteins (note there are minor differences in our estimate of the length and location of these subdomains and those of Mencarelli *et al*. [4]; Figure 2b). Nevertheless, isomin coil 1A harbors a major part of the conserved IF consensus amino acid motif 1 (IF consensus motif 1, see [8]). The central part of this motif is **LN**D**RLA**TY in the fly lamin and **LN**V**RLA**DV in isomin (common amino acids are in bold), and also the six preceding amino acids are highly homologous, indicating that they may take part in similar molecular interactions.  The second consensus motif, IF consensus motif 2, is also significantly similar: A**YD**K**L**LVG**EE**A**R** in the fly lamin and K**YD**S**L**VKV**EE**V**R** in isomin. How far this sequence has drifted from the standard IF consensus motif 2 sequence, both in isomin and in lamin Dm0, becomes clear when one compares this sequence with those present in lamins from lower invertebrates, which are nearly identical to human lamin A [1,2]. Nevertheless, this sequence homology points to a consensus that may be functionally important (see below).

**Figure 3:**
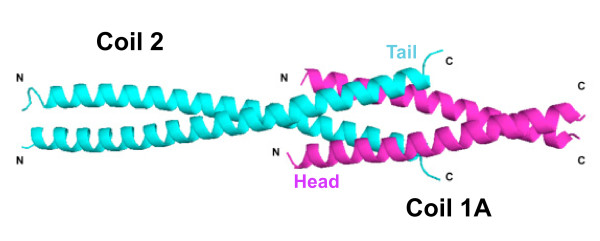
**Possible mode of IF assembly.** Hypothetic model of the 3 nm “head-to-tail” overlap of two consecutive dimers as proposed for lamin dimers (adapted from [8]). This overlap engages both IF consensus motif 1 and IF consensus motif 2 from two dimeric chains as concluded from chemical crosslinking data and measurements of lamin head-to-tail dimer chains and paracrystals (for details see [1,10]).

What may be the function of these motifs and why are they found in all IF proteins? We have recently proposed that both consensus domains are essential for IF protein assembly: specifically, that the longitudinal head-to-tail association of two dimers, leading to a structural overlap of about 3 nm, is mediated by the formation of two ‘hetero-coiled coils’ in the overlap zone (Figure 3; [8]). Such an interaction is consistent with the primary sequences observed in isomin. However, this assumes that isomin is a homopolymeric IF protein, and on the basis of the present data in Mencarelli and colleagues, other possibilities cannot be ruled out.

For example, extended keratin filaments form from obligate heterodimeric assembly pairs, yet keratin homopolymers may nonetheless form short fibrils (though not full-length ones) *in vitro*. Invertebrate cytoplasmic IF proteins often come in pairs too. Most notably, the mammalian IF multigene family harbors several members that are not able to assemble into *bona fide* IFs on their own, but require a ‘co-assembler’ partner. Nestin, synemin, syncoilin and individual type I and type II keratins are a few examples. Finally, the *in vitro* assembled filaments of isomin resemble only to some extent typical cytoplasmic IFs, especially when compared with those generated from *Ascaris suum *[9], although this may be attributable to different assembly conditions. Nevertheless, the  tissue localisation of isomin and its resistance against extraction with high salt/non-ionic detergent-containing buffers, as well as its structural organization,  all argue for its being a cytoplasmic IF-like protein.

## *And their function is …?*

In the gold-rush era of IFs, Elias Lazarides suggested in a highly cited *Nature* review that IFs serve as “mechanical integrators of cellular space”. Soon after, they were argued to be the cellular “absorbers of mechanical stress”, when it was found that mutations in epidermal keratins would lead to severe skin blistering diseases (for review see [1]). On this basis, the absence of cytoplasmic IFs from insects was plausibly explained by the presence of an exoskeleton able to protect their cells from mechanical stress. However it seems equally likely that insect cells do need proteins performing IF-like functions, and that these may, at least in some insects, be subsumed by other proteins.

For example, it has been shown that muscle cells depend on IFs for proper function: the absence of the muscle-specific desmin filaments in knockout mice is lethal if the mice are subjected to severe exercise stress, and desmin is thus likely to be essential for survival outside of the laboratory. Similarly, *Caenorhabditis elegans* needs its muscle IF protein to survive [3]. In this case, the essential property of IFs is probably elasticity - they can bear an impressive load before they break, and therefore are good candidates for contributing to the elasticity of cells. This property has been demonstrated with whole cells as well as at the individual filament level (for review see [10]). Some protein with this property is likely to be required by insects. 

Conversely, there are several IF proteins whose functions *in vivo* are not yet clear but may be quite diverse. Nestin, for example, which is used simply as a stem cell marker by most life scientists, is not able to form IFs on its own and poisons the assembly of vimentin, the hallmark IF protein of mesenchymal cells when present even in the low percent range. It has a very long tail of about 1300 amino acids with many potential interaction sites, and the sequences of this tail domain are quite different between close relatives such as man and rat, where in contrast the rod domains are more than 90% sequence identical. Hence, nestin has evolved away from the standard IF protein quite extensively, suggesting that IF proteins are easily, and in mammals frequently, adapted for new functions thereby losing their ‘standard’ molecular organization.

## *So what about isomin?*

We would suggest that isomin may have a distinct tissue-specific role in *Isotomurus* that is not necessarily restricted to generating structural order and support. For this reason, its sequence may have drifted away from the standard IF pattern, especially if isomin is in fact a ‘co-assembler’ analogous to other IF proteins such as synemin, nestin or the high molecular weight neurofilament triplet protein, with a hitherto unrecognized partner. It seems possible from this case that *Isotomurus* may harbor more IF proteins, unrecognized until now, and isomin may herald the discovery of much greater IF complexity in hexapoda. Model organisms such as *Drosophila melanogaster* may well not represent their whole phylum, and many different types of sub-cellular organization may have occurred in evolution using different proteins. This is of course particularly credible for a class of proteins that is characteristically expressed in a tissue-specific manner. A human neuronal IF protein is entirely different by sequence from an epidermal keratin, except for the existence of both IF consensus motifs and the structural organization of their α-helical segments. And indeed, the number of amino acids per α-helical segment seems not to be sacrosanct: In the *Caenorhabditis elegans* lamin, but not in that of other invertebrates, two heptads are missing in coil 2B. Hence, there is room for sequence variation and domain alterations to explore new tissue-specific functions in the course of evolution. This rather radical degree of structural flexibility is obviously a driving force in the evolution of specific tissue functions.
